# Identification and validation of an immune-related prognostic signature and key gene in papillary thyroid carcinoma

**DOI:** 10.1186/s12935-021-02066-9

**Published:** 2021-07-15

**Authors:** Rujia Qin, Chunyan Li, Xuemin Wang, Zhaoming Zhong, Chuanzheng Sun

**Affiliations:** 1 Department of Head and Neck Surgery Section II, The Third Affiliated Hospital of Kunming Medical University/Yunnan Cancer Hospital, 519 Kunzhou Road, Kunming, 650118 China; 2 Department of Medical Oncology, the First Affiliated Hospital of Kunming Medical University, 295 Xichang Road, Kunming, 650032 China

**Keywords:** Papillary thyroid carcinoma, Immune-related genes, Prognosis, PPI, Tumor infiltrating immune cells

## Abstract

**Background:**

Papillary thyroid carcinoma (PTC) is the most common pathological type of thyroid cancer. The effect of traditional anti-tumor therapy is not ideal for the patients with recurrence, metastasis and radioiodine resistance. The abnormal expression of immune-related genes (IRGs) has critical roles in the etiology of PTC. However, the effect of IRGs on PTC prognosis remains unclear.

**Methods:**

Based on The Cancer Genome Atlas (TCGA) and ImmPort databases, we integrated IRG expression profiles and progression-free intervals (PFIs) of PTC patients. First, we identified the differentially expressed IRGs and transcription factors (TFs) in PTC. Subsequently, an IRG model that can predict the PFI was constructed by using univariate Cox regression, least absolute shrinkage and selection operator (LASSO) regression and multivariate Cox regression analyses of the differentially expressed IRGs in the TCGA. Additionally, a protein–protein interaction (PPI) network showed the interactions between the differentially expressed genes (DEGs), and the top 30 genes with the highest degree were extracted from the network. Then, the key IRG was identified by the intersection analysis of the PPI network and univariate Cox regression, which was verified the differential expression of by western blotting and immunohistochemistry (IHC). ssGSEA was performed to understand the correlation between the key IRG expression level and immune activity.

**Results:**

A total of 355 differentially expressed IRGs and 43 differentially expressed TFs were identified in PTC patients. Then, eight IRGs were finally utilized to construct an IRG model. The respective areas under the curve (AUCs) of the IRG model reached 0.948, 0.820, and 0.831 at 1, 3 and 5 years in the training set. In addition, lactotransferrin (LTF) was determined as the key IRG related to prognosis. The expression level of LTF in tumor tissues was significantly lower than that in normal tissues. And the results of ssGSEA showed the expression level of LTF is closely related to immune activity.

**Conclusions:**

These findings show that the prognostic model and key IRG may become promising molecular markers for the prognosis of PTC patients.

## Background

Thyroid cancer is the most common malignancy originating from the endocrine system. The incidence rate has risen markedly in recent years [1, 2]. Papillary thyroid carcinoma (PTC) is the most common type among thyroid cancer, accounting for approximately 80–85% of reported cases [3, 4]. Most patients with PTC can be treated by surgery or radioactive iodine therapy, and the overall therapeutic effect is satisfactory [5]. However, such treatments cannot completely alleviate radiation-resistant PTC [6]. In addition, some PTC patients have a high metastasis rate and recurrence rate after conventional treatment. The most common method of metastasis is via the cervical lymph system, and metastasis is often a factor of a poor prognosis of PTC [7]. The current treatments are inadequate for PTC patients with local or distant metastasis and recurrence. Therefore, it is urgent to explore early diagnosis and intervention methods for PTC and provide personalized treatment.

It has been reported that the number and distribution of tumor-infiltrating immune cells (TICs) can affect the treatment response of tumor patients. TICs have become a promising target for further improving the prognosis of patients. Prospective immunotherapies also provide an alternative treatment option for PTC patients. Exploration of the pattern of the tumor microenvironment is helpful for judging the prognostic value and therapeutic effect of PTC patients. Immunotherapy represented by immune checkpoint inhibitors is changing the status quo of cancer treatment [8]. Programmed cell death 1 and its ligand (PD-1/PD-L1) and cytotoxic T lymphocyte associated antigen 4 (CTLA-4) are the most commonly used targeted immune checkpoints in clinical trials. They have achieved considerable efficacy in the treatment of multiple tumor types [9–11]. In addition, Bai Y et al. found a positive correlation between BRAF V600E and PD-L1/PD-1 expression in PTC patients, suggesting that immunotherapy for PD-L1/PD-1 may be effective for PTC patients with BRAF V600E mutation, and these patients were refractory to radioiodine therapy [12]. Treatment with a combination of anti-PD-1/PD-L1 and BRAF inhibitors significantly reduced the tumor volume in a mouse model of thyroid cancer [13]. Moreover, the association between PD-L1 and disease-free survival is strong in PTC, which highlighted the role of PD-L1 as a potential prognostic biomarker for disease recurrence in patients with PTC [14]. Although these findings show that immunotherapy plays a vital role in PTC, its molecular mechanism is still unclear. Currently, we can detect the abnormal expression of immune-related genes (IRGs) during tumor progression based on sequencing technology, providing effective targets for diagnosis and treatment. Therefore, it is essential to comprehensively demonstrate the therapeutic and prognostic significance of IRGs and carry out individualized immunotherapy to improve the prognosis of PTC patients.

The main aim of our study was to explore the potential prognostic values of IRGs in PTC by integrating clinical data and corresponding gene expression downloaded from The Cancer Genome Atlas (TCGA) and ImmPort databases. First, we identified the differentially expressed IRGs and transcription factors (TFs) in PTC. Subsequently, an IRG model that can predict the progression-free interval (PFI) was established by using univariate Cox regression analysis, least absolute shrinkage and selection operator (LASSO) regression analysis and multivariate Cox regression analysis from the differentially expressed IRGs in the TCGA. Receiver operating characteristic (ROC) curves were generated to analyze the specificity and sensitivity of the prognostic model. Additionally, a protein–protein interaction (PPI) network showed the interaction between the differentially expressed genes (DEGs), and the top 30 genes with the highest degree were extracted from the network. Then, lactotransferrin (LTF) was determined as a key gene related to prognosis according to the intersection analysis of the PPI network and univariate Cox regression analysis. The differential expression of LTF was verified at the cellular and tissue levels by western blotting and immunohistochemistry (IHC). These findings indicate that the prognostic model and key IRG may become promising molecular markers and provide targets for the diagnosis and prognosis of PTC.

## Materials and methods

### Data collection and preprocessing

The normalized RNA-seq data (FPKM) and corresponding clinical features of PTC patients were downloaded from the TCGA (https://portal.gdc.cancer.gov/) [15]. In addition, 2498 IRGs were downloaded from the ImmPort database (https://www.immport.org/shared/genelists, May 29, 2020), and 318 TFs were obtained from the Cistrome database (http://cistrome.org/CistromeCancer/CancerTarget/). To ensure that only significantly expressed genes were evaluated, genes with an average expression value of less than 0.1 were excluded from each sample. The DEGs between tumor samples and normal samples were determined using the Wilcoxon test method. The log2-fold change cutoff was set as 2, and the false discovery rate (FDR) cutoff was set as 0.05, and genes that met these criteria were selected as statistically significant.

### Functional enrichment analysis

We identified differentially expressed IRGs and TFs based on the differential analysis of tumor and normal samples. Then, we used the R package clusterProfiler to perform functional enrichment analysis on the IRGs and TFs [16]. The Gene Ontology (GO) terms were obtained genes with p- and q-values strictly less than 0.05. Subsequently, we explored the enriched pathways of the differentially expressed IRGs and TFs through Kyoto Encyclopedia of Genes and Genomes (KEGG) analysis. The R “GOplot” package was utilized to visualize the most significantly enriched GO terms and KEGG pathways [17]. In addition, we downloaded Gene Set Enrichment Analysis (GSEA) software from the Broad Institute (http://software.broadinstitute.org/gsea/msigdb). C7 gene set v6.2 collections and hallmarks were obtained from the Molecular Signatures Database (MSigDB) as reference gene sets. All PTC patients in the TCGA database were divided into a high LTF expression group and a low LTF expression group based on the median expression value, this was analyzed as the phenotype. The permutation number was set at 1000. A nominal p-value (NOM p) < 0.05 and FDR < 0.25 were used as the cutoff criteria to screen statistically significant pathways.

### Construction and identification of the prognostic model

To confirm the potential prognosis-related IRGs, we first analyzed the relationship between the expression of the IRGs and the PFI by performing univariate Cox regression analysis. In addition, genes that were significantly related to the PFI (p < 0.05) were selected as prognosis-related IRGs in PTC. Then, the LASSO regression approach was conducted to obtain the optimal IRGs [18]. Finally, we applied multivariate Cox regression analysis, identified eight prognosis-related IRGs and then constructed an eight-gene signature. In the training set and the whole set, we calculated the risk score of each patient based on the regression coefficient of the IRGs in the signature and the corresponding expression value of the IRG. The risk score was calculated using the following formula:

$${\text{Risk score }} = {\text{ expression of Gene }}1 * \beta 1{\text{ }} + {\text{ expression of Gene }}2 * \beta 2{\text{ }} + \ldots {\text{expression of Gene }}n * \beta n,$$

where β represents the regression coefficient of the IRGs in the signature. PTC patients in the TCGA database were divided into a high-risk group and a low-risk group based on the median risk value of the training set. Survival analysis was carried out to compare the PFIs between the high-risk group and low-risk group. The difference in PFIs and the significance of prognosis between the high- and low-risk groups were evaluated. A p-value < 0.05 was selected as the significant cutoff value. Additionally, ROC curves were utilized to evaluate the accuracy of the prediction model.

### Comprehensive analysis of the prognostic model

Univariate Cox regression analysis was conducted to evaluate the prognostic relevance of the risk model in the whole set, which included age, gender, T stage, N stage, M stage, TNM stage, tumor burden and focus type. We then evaluated the independent prognostic ability of the risk score by performing a multivariate analysis. Subsequently, we explored the correlation between the risk score and clinicopathological features to better evaluate the role of the prognostic model in the PTC development. In this study, the survival and rms packages in R were utilized to build a nomogram that included each IRG in the model. Then, calibration curves were plotted to assess the accuracy of the prediction model. In addition, according to the expression levels of IRGs in the model, two-dimensional principal component analysis (PCA) and three-dimensional PCA were carried out to explore the differences in the distribution of the low-risk group and high-risk groups.

### PPI network construction

A PPI network between DEGs was constructed based on the Search Tool for the Retrieval of Interacting Genes/Proteins (STRING) database (http://string-db.org) [19]. Nodes with interaction scores > 0.92 were considered meaningful and extracted. The data obtained from the STRING database were then imported into Cytoscape (http://cytoscape.org/). The network was visualized with the software [20]. Because TFs are considered to be vital molecules that can directly regulate the expression of IRGs, we also built and visualized the regulatory network of differentially expressed TFs and prognosis-related IRGs.

### Identification and verification of the key IRG related to prognosis

LTF was identified as the key prognosis-related IRG by combining the PPI network and univariate Cox regression analysis. First, the Tumor Immunity Estimation Resource (TIMER, https://cistrome.shinyapps.io/timer/) database was used to verify the difference in the expression level of LTF between tumor and normal tissues in multiple cancer types. Then, Kaplan–Meier (KM) curve and ROC curve analyses were carried out to evaluate the prognostic and diagnostic values of LTF. UALCAN (http://ualcan.path.uab.edu/), a web tool, was used to detect the methylation status [21].

### Evaluation of immune infiltration

Single-sample gene set enrichment analysis (ssGSEA) was conducted by utilizing the “gsva” package in R software, and the infiltration scores of 16 immune cells and the activity of 13 immune-related pathways were calculated [22]. Subsequently, we explored the correlation between the expression level of LTF and immune cell infiltration and immune-related pathways.

### Cell culture

The normal thyroid follicular epithelial cell line Nthy-ori 3–1 was provided by the Institute of Medical Biology Chinese Academy of Medical Sciences (Kunming), and the PTC cell lines K1, BCPAP and TPC-1 were obtained from Sun Yat-sen University Cancer Center (Guangzhou). All the cell lines were cultured in Dulbecco's modified Eagle's medium (DMEM) supplemented with 10% fetal bovine serum (FBS) (Gibco) at 37 °C in a humidified 5% CO_2_ atmosphere. All cell lines were proven to be mycoplasma negative.

### Western blotting

Total proteins were extracted from all cell lines. The protein concentration was detected with the BCA protein assay. Then, 30 μg total protein samples were subjected to SDS-PAGE, and the separated bands were transferred to 0.22 μm PVDF membranes. Protein was blocked for 1 h with blocking solution. The membrane was incubated with the primary antibody overnight at 4 °C and with the secondary antibody at room temperature for 1 h. Finally, the gel was imaged. Anti-LTF and anti-GAPDH antibodies were purchased from Proteintech. In addition, GAPDH served as the loading control for all samples.

### IHC

To verify the expression of LTF in PTC and adjacent normal tissues, we conducted experimental validation in 30 samples of PTC patients who underwent total thyroidectomy in the Third Affiliated Hospital of Kunming Medical University. The study was reviewed and approved by the Third Affiliated Hospital of Kunming Medical University. The paraffin-embedded tumor tissues and adjacent tissues of PTC patients were collected. Immunohistochemical staining was performed with the anti-human LTF antibody (1:100, 10933-1-AP, Proteintech). Slides were incubated with primary antibody at 4 °C overnight, followed incubation with secondary antibody for 30 min at 37 °C. Slides were then immersed in 3.3’-diaminobenzidine and counter-stained with 10% Mayer’s hematoxylin, dehydrated, and mounted. The percentage of positive staining (0, 0–5%; 1, 6–25%; 2, 26–50%; 3, 51–75%; and 4, 76–100%) and the staining intensity (0, negative; 1, weak; 2, moderate; and 3, strong) were recorded. IHC results were evaluated by two experienced pathologists independently.

### Statistical analyses

All statistical analyses were managed by R software (Version 3.6.3) and SPSS (Version 25). Student’s t-test was used for statistical comparisons, and p < 0.05 was selected as statistically significant.

## Results

### Identification of the differentially expressed IRGs and TFs in PTC patients

**Fig. 1:**
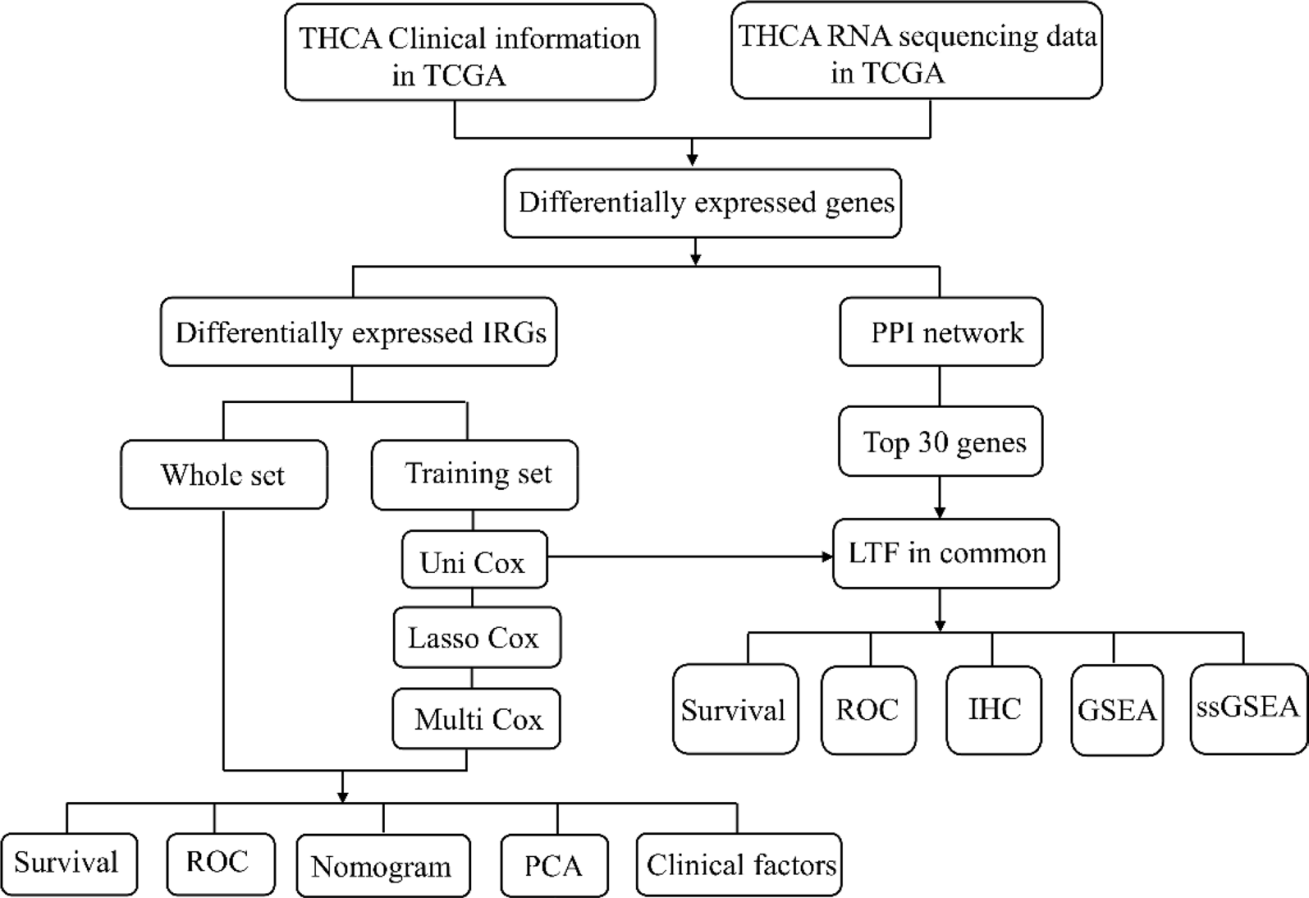
Flow chart of the analysis process in our study

**Fig. 2:**
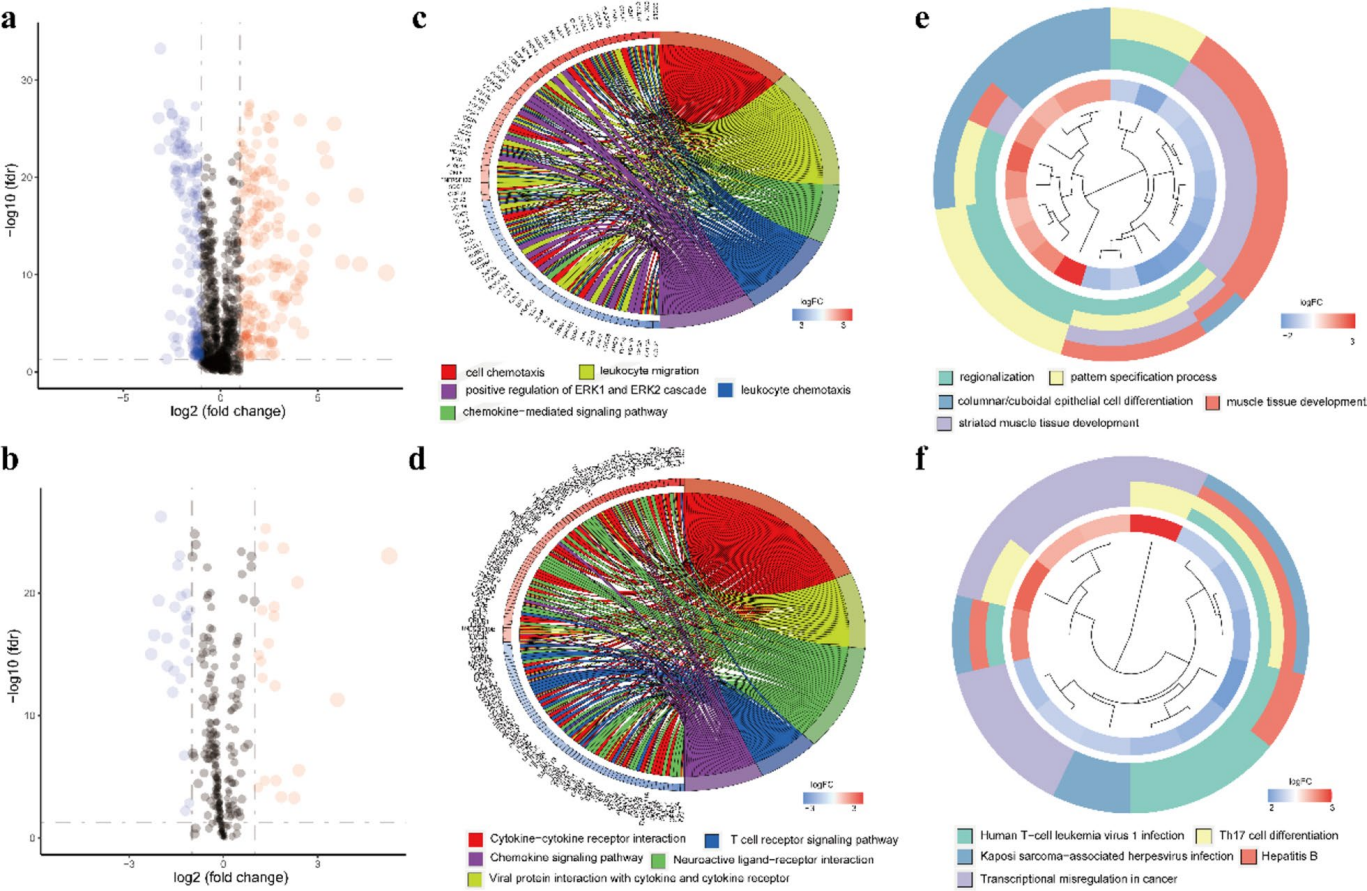
Differentially expressed IRGs and TFs between PTC and normal thyroid samples and functional enrichment analysis. **a**, **b** Volcano plots of the differentially expressed IRGs and TFs. **c**,** d** GO enrichment analysis and **e–f** KEGG enrichment analysis of the differentially expressed IRGs and TFs

The general analysis process of this study is displayed in Fig. 1. We obtained RNA-sequencing data and clinical follow-up data of 493 PTC samples and 58 normal thyroid tissue samples from the TCGA dataset. IRGs are usually regulated by TFs and play a pivotal role in the tumor microenvironment. Then, 2498 IRGs were downloaded from the ImmPort database, and 318 TFs were downloaded from the Cistrome database. By comparing PTC samples and normal samples from the TCGA database, we identified 1648 DEGs and then screened 355 differentially expressed IRGs and 43 differentially expressed TFs from the DEGs, and the data are displayed as a volcano map (Fig. 2a, b).

To further explore the potential mechanisms and biological functions of the DEGs, GO and KEGG pathway enrichment analyses were carried out on the differentially expressed IRGs and TFs. Regarding the GO analysis, IRGs were mostly enriched in cell chemotaxis, leukocyte migration and the chemokine-mediated signaling pathway (Fig. 2c). In addition, TFs were significantly enriched in the cytokine-cytokine receptor interaction, viral protein interaction with cytokine and cytokine receptor and chemokine signaling pathway (Fig. 2d). In the KEGG pathway analysis, the differentially expressed IRGs were mainly associated with regionalization, pattern specification process and striated muscle tissue development (Fig. 2e), and the differentially expressed TFs were mainly enriched in human T-cell leukemia virus 1 infection, Th17 cell differentiation and transcriptional misregulation in cancer (Fig. 2f).

### Construction of the prognostic risk model and analysis

**Fig. 3:**
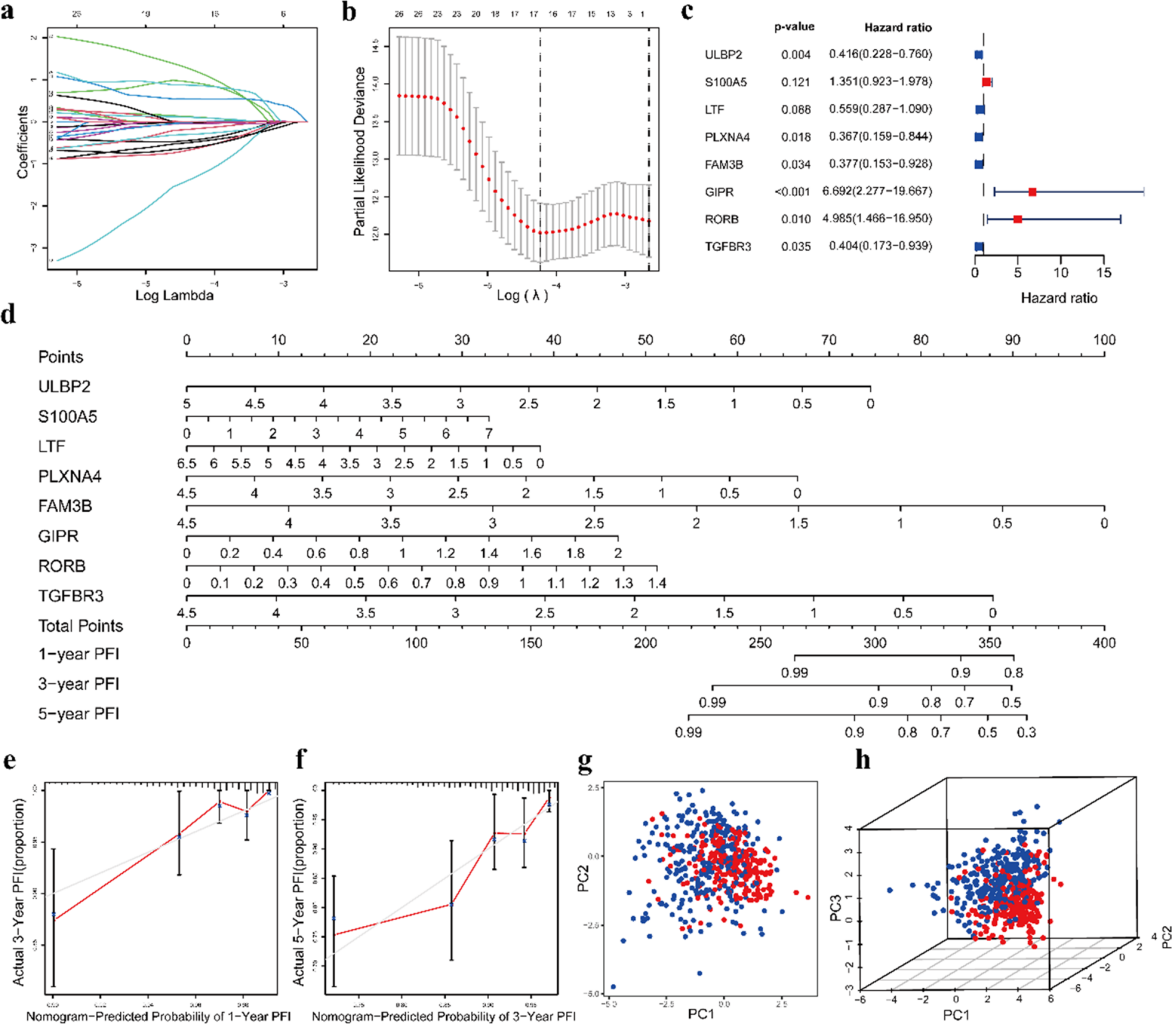
Construction of the prognostic risk model and analysis. **a**, **b** LASSO regression analysis of the PFI-associated IRGs. **c** The hazard ratios and p-values from the multivariate Cox regression are shown in the forest plot. **d** Nomogram showing the PFIs at 1, 3 and 5 years of patients in the TCGA database. **e**, **f** Calibration curves of the nomogram to predict the PFIs at 1 and 3 years. **g**, **h** Two-dimensional PCA plot and three-dimensional PCA plot showing distribution in the high-risk group and low-risk group

To further clarify the correlation between the IRGs and prognosis, we constructed an eight-gene model based on the IRGs to predict the progression and survival of PTC patients. First, we randomly divided the whole dataset containing survival information into a training set and a test set at a ratio of 1:1. Twenty-seven IRGs were significantly associated with PFIs in the training set according to the univariate Cox regression analysis. IRGs related to PFIs were subsequently subjected to LASSO regression analysis to improve the prognostic ability of the model (Fig. 3a, b). Ultimately, a prognostic signature comprising eight IRGs, namely, UL16 binding protein 2 (ULBP2), S100 calcium binding protein A5 (S100A5), LTF, plexin A4 (PLXNA4), FAM3 metabolism regulating signaling molecule B (FAM3B), gastric inhibitory polypeptide receptor (GIPR), RAR related orphan receptor B (RORB) and transforming growth factor beta receptor 3 (TGFBR3), was selected to build a prognostic signature by stepwise multivariate Cox regression analysis (Table 1), and the forest plot is shown in Fig. 3c. The risk score was calculated based on the following equation:

$$\begin{aligned} {\text{Risk score}} &= \left( { - 0.{\text{876}}*{\text{ULBP2}}} \right) + \left( {0.{\text{3}}0{\text{1}}*{\text{S1}}00{\text{A5}}} \right) \\ &\quad+ ( - 0.{\text{582}}*{\text{LTF}}) + \left( { - {\text{1}}.00{\text{3}}*{\text{PLXNA4}}} \right) \\ &\quad + \left( { - 0.{\text{975}}*{\text{FAM3B}}} \right) + \left( {{\text{1}}.{\text{9}}0{\text{1}}*{\text{GIPR}}} \right){\text{ }} \\ & \quad + \left( {{\text{1}}.{\text{6}}0{\text{6}}*{\text{RORB}}} \right) + \left( { - 0.{\text{9}}0{\text{7}}*{\text{TGFBR3}}} \right). \\ \end{aligned}$$

**Table 1:** Multivariate Cox regression analysis for PFI of eight IRGs in PTC

ID	Coefficient	HR	HR.95L	HR.95H	p-value
ULBP2	− 0.876	0.416	0.228	0.760	0.004
S100A5	0.301	1.351	0.923	1.978	0.121
LTF	− 0.582	0.559	0.287	1.090	0.088
PLXNA4	− 1.003	0.367	0.159	0.844	0.018
FAM3B	− 0.975	0.377	0.153	0.928	0.034
GIPR	1.901	6.692	2.277	19.667	0.001
RORB	1.606	4.985	1.466	16.950	0.010
TGFBR3	− 0.907	0.404	0.173	0.939	0.035

*HR,* Hazard ratio

Nomograms play an integral part in the decision-making process of modern medicine because they can help predict the likelihood of clinical events by using different prognostic factors and determinants [23]. To provide a quantitative approach to evaluate the PFIs of PTC patients, we built a nomogram based on risk scores and an eight-gene marker. The nomograms of the 1-, 3-, and 5-year PFIs of PTC patients are shown in Fig. 3d. The calibration curve showed that the 1- and 3-year PFIs predicted by the nomogram were very consistent with the actual observations, indicating that the nomogram was accurate (Fig. 3e, f). Then, all PTC patients in the TCGA database were divided into a high-risk group and a low-risk group according to the median cutoff value. Subsequently, we conducted PCA to explore the respective distribution between the high- and low-risk groups. The two-dimensional PCA and three-dimensional PCA showed that patients in the high- and low-risk groups were significantly distributed on both sides (Fig. 3g, h).

### Validation of the prognostic risk model

**Fig. 4:**
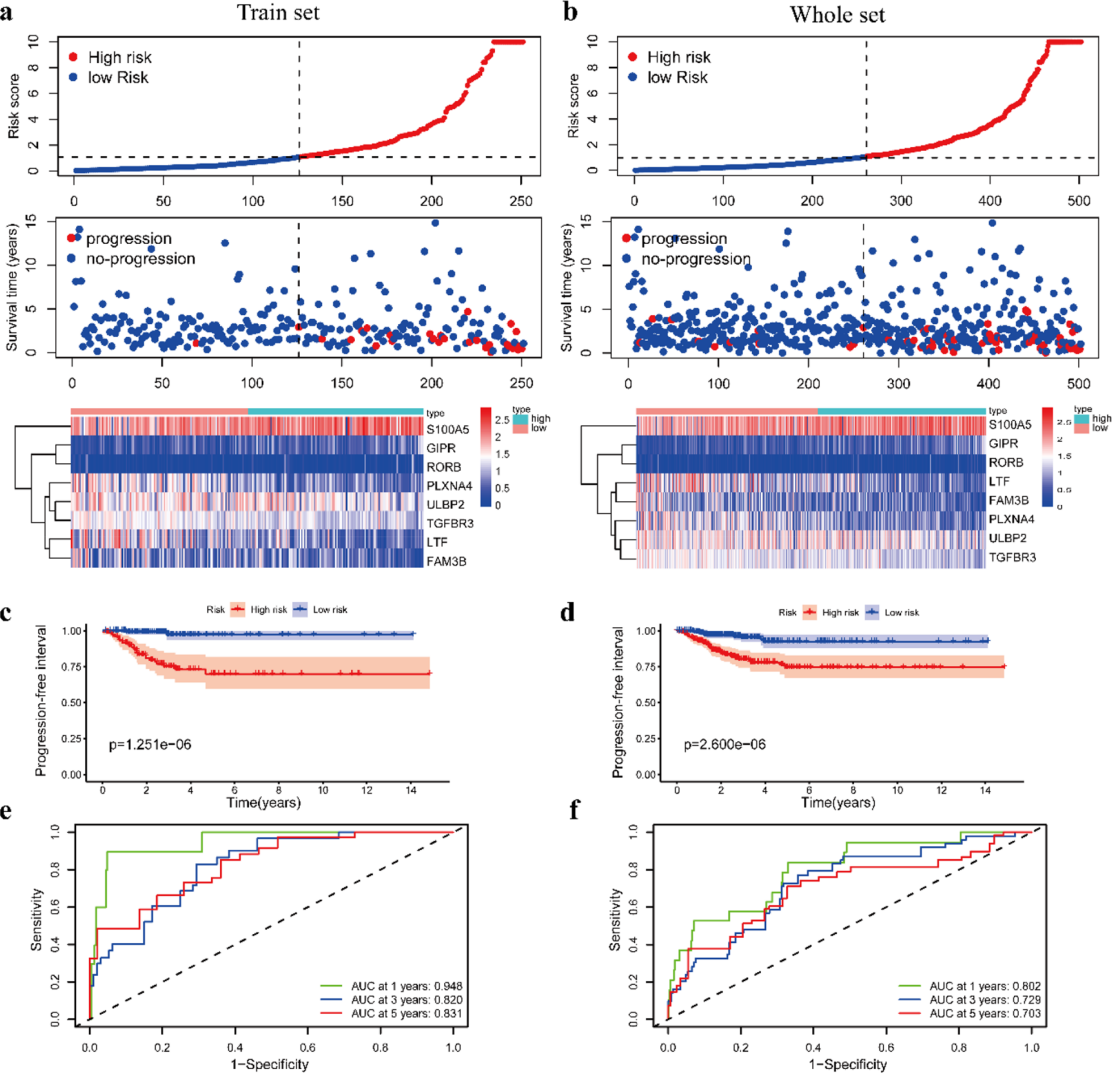
Prognostic risk model in PTC patients from the training set and the whole set. **a**, **b** Patients ranked by risk score, corresponding survival status and heatmap of the training set and the whole set. **c**, **d** Kaplan–Meier survival curve of PFIs of PTC patients in the training set and the whole set according to the median cutoff value. **e**, **f** ROC curves at 1, 3, and 5 years in the training set and the whole set

The PTC patients were divided into a low-risk group and a high-risk group according to the cutoff value, and then we calculated the prognostic risk score of each PTC patient. The distribution of the risk score, survival status and corresponding heatmap of the expression level of IRGs in patients in the training set and the whole set are displayed in Fig. 4a, b. To evaluate the impact of a high-risk score and low risk score on prognosis, we evaluated the PFIs in the TCGA data by performing KM curve analysis. The results are shown in Fig. 4c, d. The prognosis of PTC patients in the low-risk group was better than that of patients in the high-risk group (p < 0.001). To further clarify the accuracy of the eight-IRG model to predict the PFIs of PTC patients, we analyzed the time-dependent ROC curves. In the training set, the respective areas under the curve (AUCs) of the prognostic signature reached 0.948, 0.820, and 0.831 at 1, 3 and 5 years, respectively. Similarly, in the whole set, the AUCs were 0.802, 0.729, and 0.703, respectively (Fig. 4e, f). In general, these results indicate that the eight-IRG model has good accuracy in predicting the occurrence and development of PTC.

**Fig. 5:**
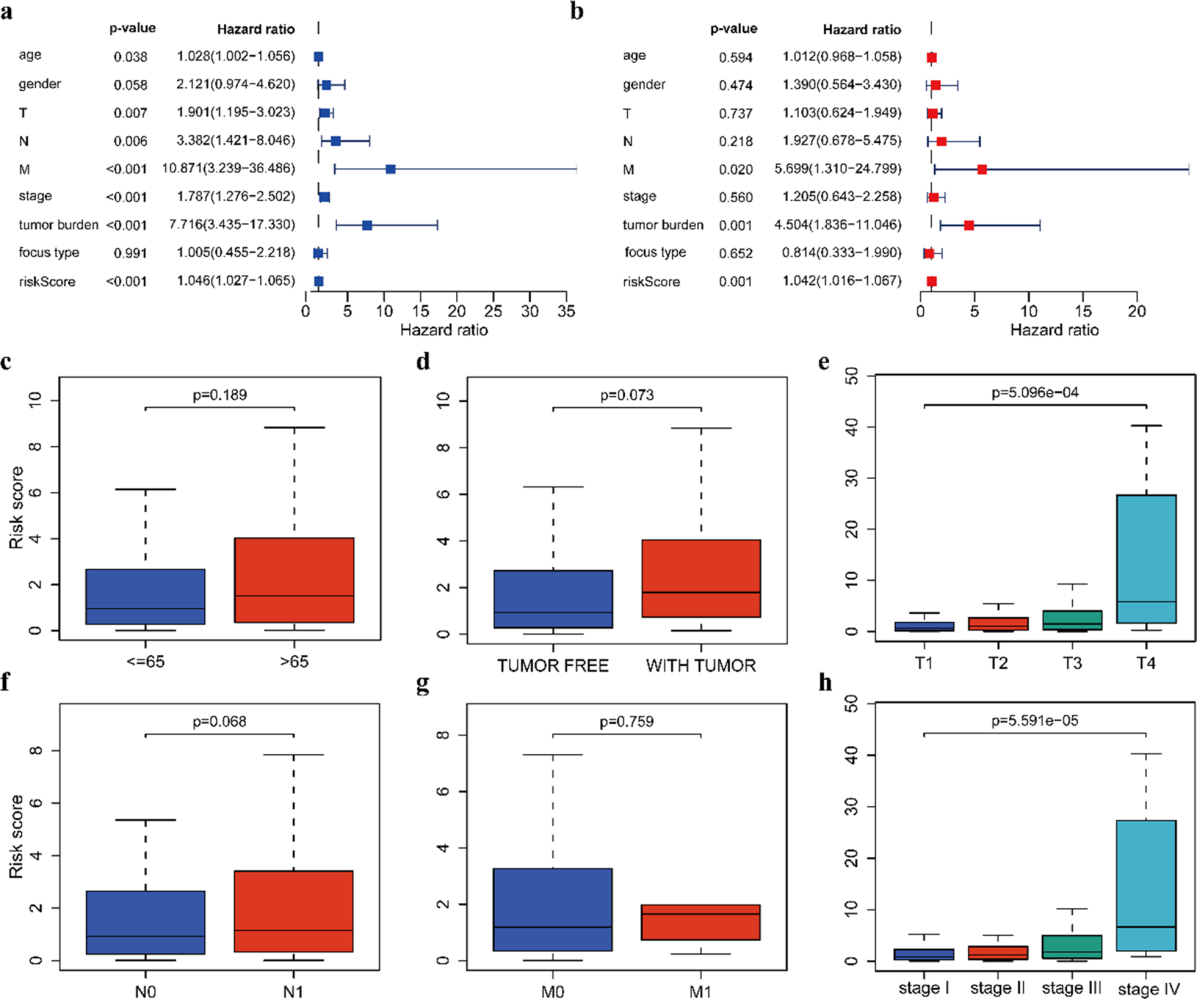
Analysis of the prognostic risk model. **a**, **b** Univariate Cox regression analysis and multivariate Cox regression analysis of clinical parameters and the risk score in the whole set. **c–h** Correlation of the risk score with age, tumor burden, T stage, N stage, M stage and TNM stage of PTC

To evaluate the independent prognostic ability of the prognostic model, we carried out univariate and multivariate Cox regression analyses on TCGA data. In the univariate Cox regression analysis, the risk score was related to the PFI in PTC patients (Fig. 5a). In the multivariate Cox regression analysis, the risk score also had a certain predictive value for the PFI (Fig. 5b). These findings illustrate that the risk score based on eight IRGs can be considered an independent prognostic factor for survival in PTC patients. Subsequently, we explored the clinical relevance of our eight-IRG signature and evaluated the correlation between the risk score and clinicopathological parameters of PTC patients in the whole set. The results showed that our eight-IRG signature was significantly related to T stage (p < 0.001) and TNM stage (p < 0.001) (Fig. 5c–h). As the risk score increased, the probability of progressing to an advanced tumor gradually increased, suggesting that our IRG signature may play a pivotal role in the progression of PTC.

### Intersection analysis of the PPI network and univariate Cox regression Analysis

**Fig. 6:**
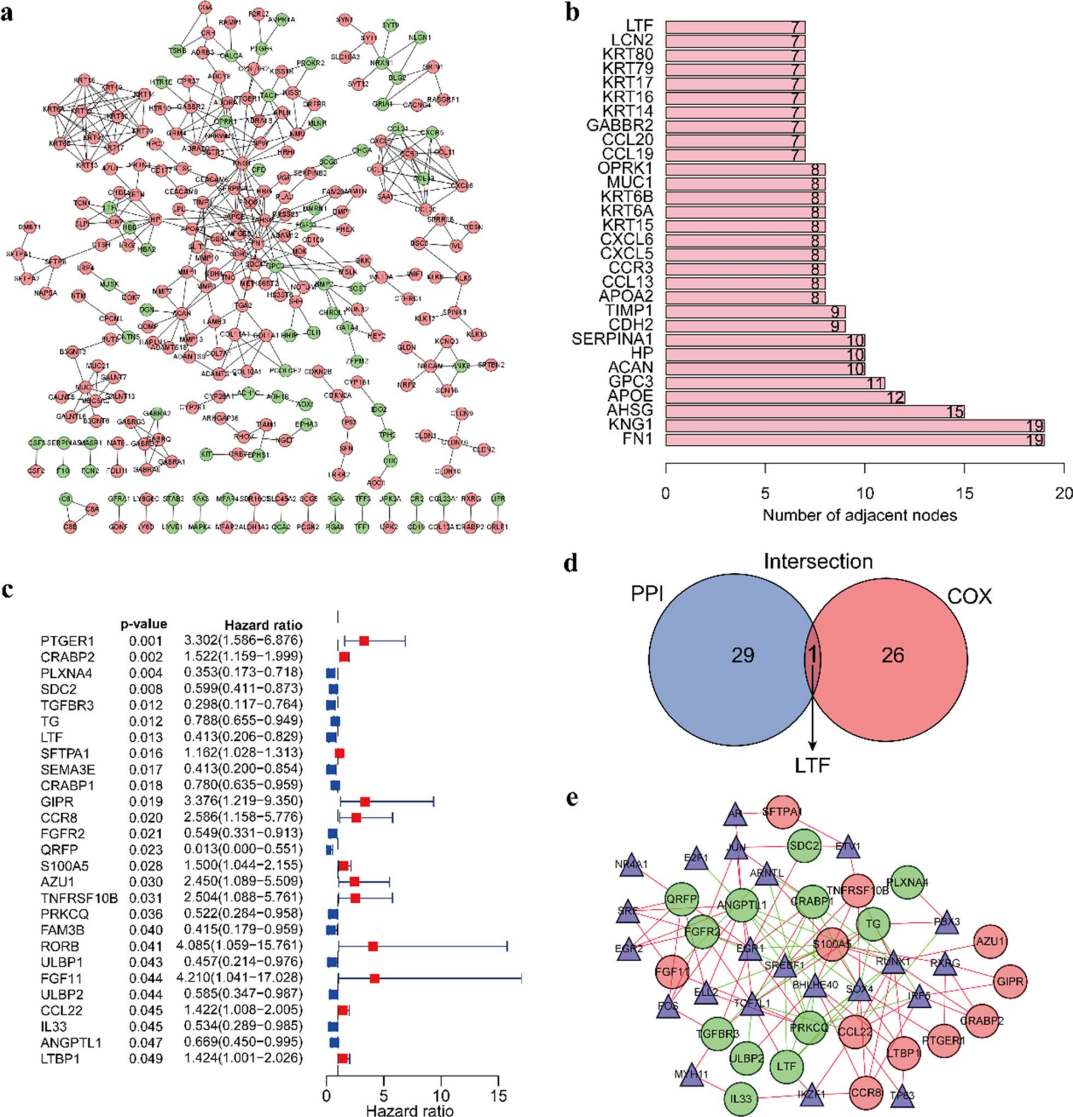
PPI network and univariate Cox regression analysis. **a** PPI network of the DEGs. **b** Bar plot showing the top 30 genes ordered by the number of nodes. **c** Forest plot showing the prognosis-related IRGs screened by the univariate Cox regression analysis. **d** Venn diagram displaying the common genes shared by the top 30 nodes in the PPI network and prognosis-related IRGs. **e** Interaction network between TFs and prognosis-related IRGs. Triangles: TFs; circles: IRGs; red circles: IRGs that positively correlated with PFIs; green circles: IRGs that negatively correlated with PFIs; green line and red line indicate a negative correlation and positive correlation, respectively

To identify the potential interaction network between DEGs, we used Cytoscape software to build a PPI network based on the STRING database, integrating 268 nodes and 405 edges (Fig. 6a). The bar plot shows the top 30 genes ordered by the number of nodes (Fig. 6b). A total of 27 IRGs were significantly correlated with the PFIs by the univariate Cox regression analysis based on the DEGs. The forest plot is shown in Fig. 6c. Then, intersection analysis of the top 30 nodes of the PPI network and the prognosis-related IRGs filtered by univariate Cox regression was carried out (P < 0.05). In the above analysis, only one factor, LTF, overlapped and was identified as the key IRG related to prognosis (Fig. 6d). TFs are considered to be vital molecules that can directly regulate the expression of other genes. Therefore, we explored the underlying interaction between the differentially expressed TFs and prognosis-related IRGs screened by univariate Cox regression. The interaction network between TFs and IRGs is shown in Fig. 6e.

### Analysis and validation of LTF expression in PTC patients

**Fig. 7:**
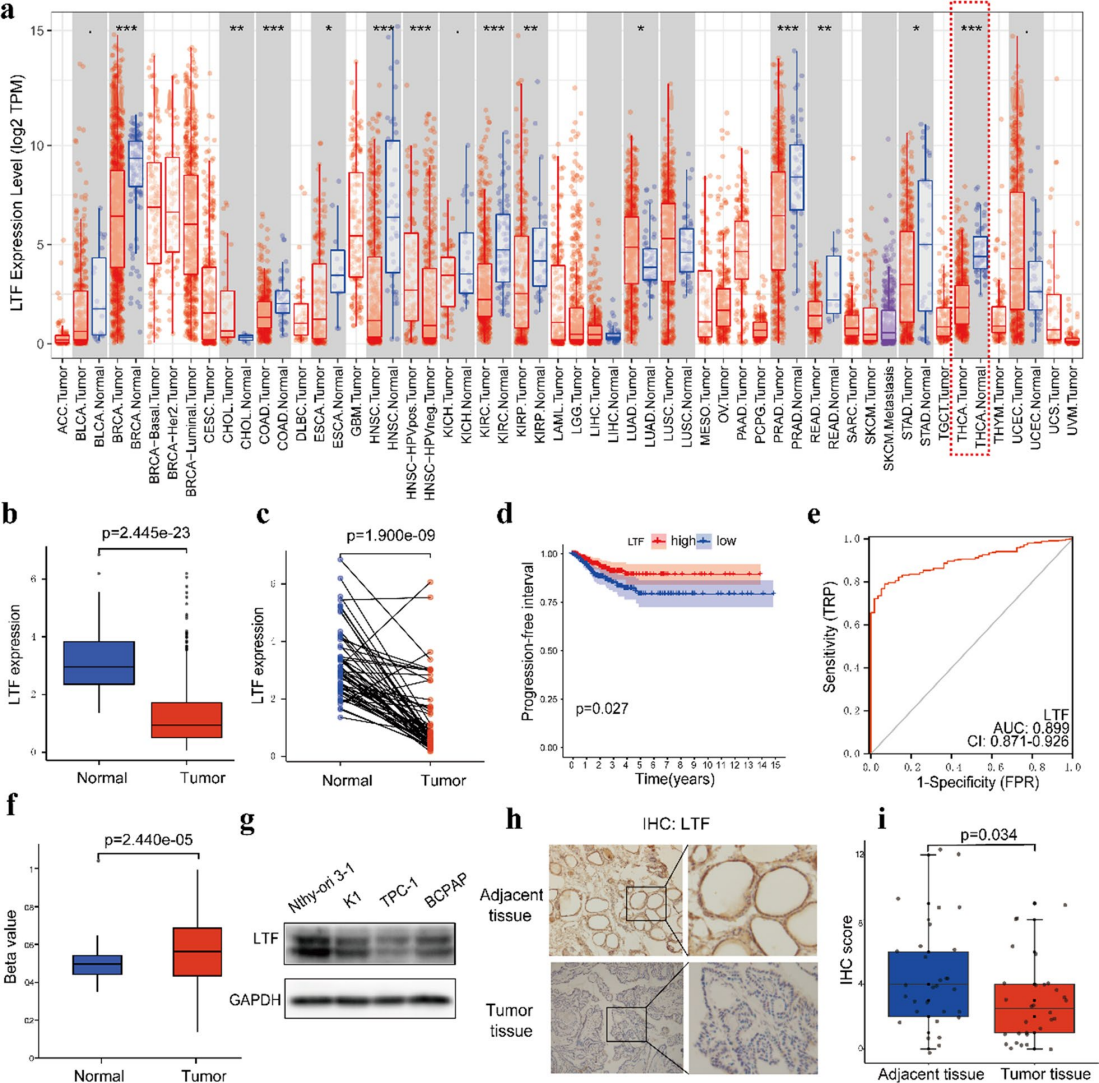
The differential expression of LTF and its association with survival and potential functional mechanism in PTC patients. **a** LTF expression in multiple tumor and normal tissues based on the TIMER database. **b** Differentiated expression of LTF in tumor and normal tissues from the TCGA database. **c** Paired analysis of LTF expression between tumor and normal tissues from the same patient in the TCGA database. **d** KM survival curve of PFIs in patients in the low LTF and high LTF expression groups in the TCGA database. **e** Diagnostic efficacy of the ROC curve of LTF. **f** Methylation level of LTF according to UALCAN. **g** LTF protein expression levels in the normal thyroid follicular epithelial cell line Nthy-ori 3–1 and PTC cell lines. **h** Examples of IHC staining of LTF in PTC tissues and adjacent noncancerous tissues. **i** Comparison of LTF protein expression in 30 pairs of matched paraffin section samples by IHC

**Fig. 8:**
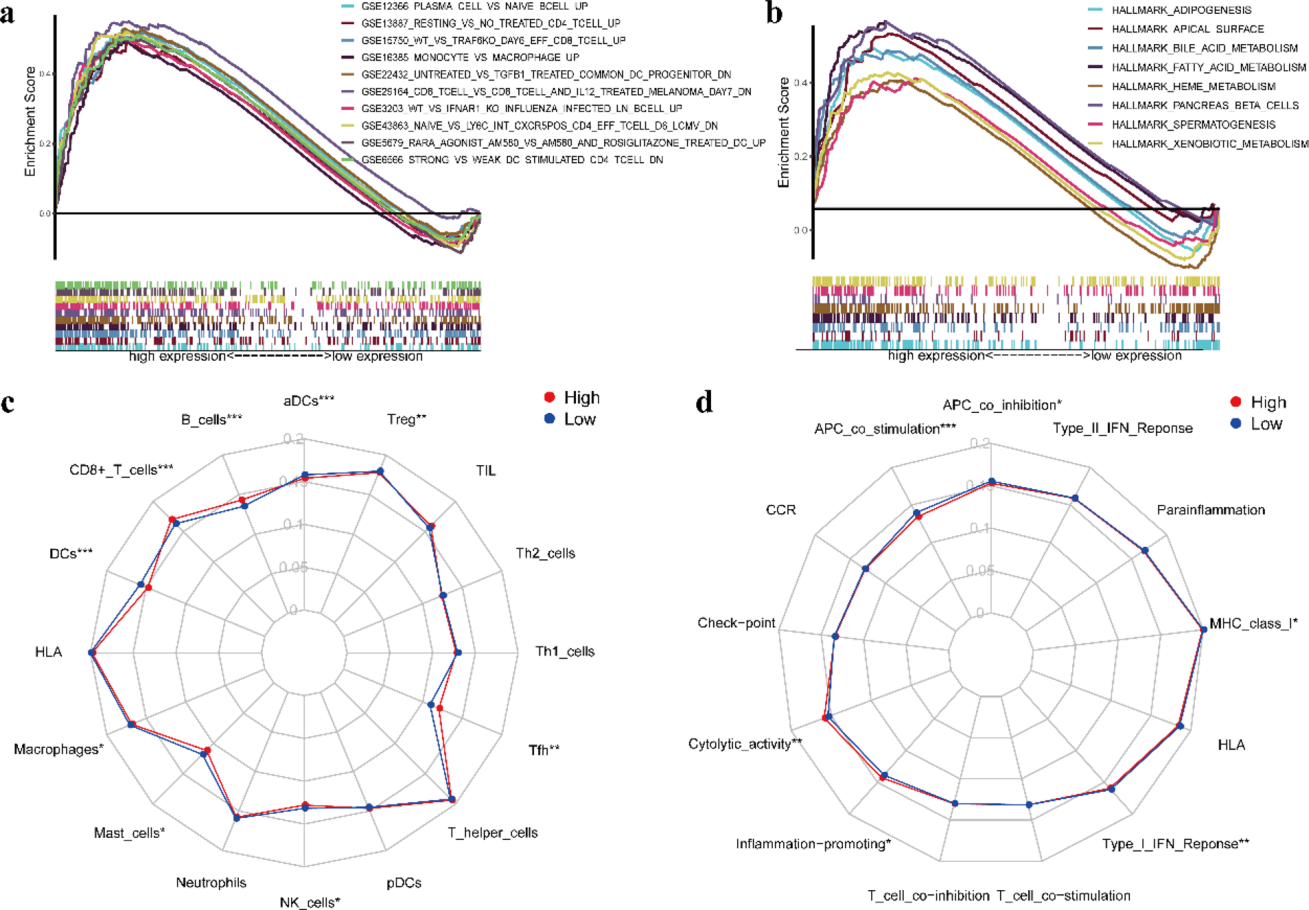
GSEA and ssGSEA scores between the different LTF expression groups. **a** Enriched gene sets in the C7 collection in the high LTF expression group. **b** Enriched gene sets in the HALLMARK collection in the high LTF expression group. **c** Boxplots showing the scores of 16 immune cells in the different LTF expression groups. **d** Boxplots showing the scores of 13 immune-related functions in the different LTF expression groups. The p-values were uniformly replaced with the following symbols: *p < 0.05; **p < 0.01; ***p < 0.001

LTF is a vital component of the nonspecific immune system. It plays an important role in tumor progression [24, 25]. First, we used the TIMER database to examine LTF expression in multiple tumors, including PTC. The results showed that the expression of LTF in tumor tissues was significantly lower than that in normal tissues (Fig. 7a). Then, we analyzed data from the TCGA and obtained the same result (Fig. 7b). The paired differentiation analysis indicated that the LTF expression level was also lower in tumor tissues than in normal tissues from the same patient (Fig. 7c). To evaluate the prognostic value of LTF in PTC, KM curve analysis was carried out, which indicated that patients with low LTF expression levels had shorter progression‐free survival times than those with high LTF expression levels (Fig. 7d). Tumor and normal thyroid tissues were distinguished by ROC curve analysis, and we found that the AUC of the LTF expression level was 0.899, suggesting that it may be a good diagnostic biomarker (Fig. 7e). It has been reported that LTF has a high methylation level in tumors [26, 27]. To elucidate the potential mechanism of LTF downregulation in PTC, UALCAN analysis indicated that the methylation level of LTF in tumor tissues was significantly higher than that in normal tissues (Fig. 7f). In addition, we verified the expression of LTF at the cellular level and found that the expression level of LTF in PTC cell lines was significantly lower than that in the normal thyroid follicular epithelial cell line Nthy-ori 3–1 (Fig. 7g). To further verify the expression of LTF at the tissue level, IHC analyses were carried out to compare the expression level of LTF in human PTC and adjacent noncancerous tissue. The examples of IHC staining were shown in Fig. 7h. The results indicated that the expression of LTF in tumor tissues was significantly lower than that in adjacent tissues (Fig. 7i). In addition, considering the negative correlation between LTF expression and PFI and TNM stage in patients with PTC, we used GSEA to identify the enriched features and functional differences between the high LTF and low LTF expression groups. The high LTF expression group was mainly enriched in “ADIPOGENESIS”, “APICAL_SURFACE”, “BILE_ACID_METABOLISM”, and “FATTY_ACID_METABOLISM”, as displayed in Fig. 8b. Regarding the C7 collection defined by the MSigDB, the immunologic gene sets and genes in the high LTF expression group showed enrichment in “PLASMA_CELL_VS_NAIVE_BCELL_UP”, “RESTING_VS_NO TREATED_CD4_TCELL_UP”, and “MONOCYTE_VS_MACROPHAGE_UP” (Fig. 8a). Therefore, the GSEA findings imply that immune-related signals are correlated with the occurrence and development of PTC.

### ssGSEA

With in-depth research on immunotherapy, emerging research supports the crucial role of the tumor microenvironment in the response to immunotherapy [28]. The tumor microenvironment has completely distinct functions in different stages of tumor development. To further understand the correlation between the LTF expression level and immune activity, we calculated the enrichment score of different immune cell subsets, as well as the immune-related functions and pathways based on the ssGSEA algorithm. Interestingly, nine kinds of immune cells had a significant correlation with LTF expression: macrophages, mast cells, natural killer (NK) cells, Tfh cells, activated dendritic cells (aDCs), B cells, Tregs, CD8 + T cells and DCs (Fig. 8c). Six immune-related functions and pathways were significantly related to LTF expression: APC coinhibition, APC costimulation, cytolytic activity, inflammation promoting, type I IFN response and MHC class I (Fig. 8d). The ssGSEA results further verified that the LTF expression level may influence the immune status of the tumor microenvironment.

## Discussion

PTC is the most common pathological type of thyroid cancer. PTC patients have a better prognosis than patients with other malignant tumors. However, many patients with PTC experience local and distant metastasis, and the recurrence rate is still as high as 30%. Although the effect of traditional antitumor therapy is relatively ideal. However, there are still a small number of PTC patients resistant to traditional treatment, especially the patients who can not be operated or who have recurrence after operation and who have no response to iodine therapy. The traditional antitumor therapy can not solve all the problems of PTC patients [29, 30]. Therefore, it is necessary to develop new molecular targets to monitor the therapeutic effect and predict the progression of PTC to help improve patient care.

Immunotherapy is a vital treatment method for a variety of tumors. IRGs in tumors are closely correlated with tumor progression [31, 32]. At present, the patients with recurrence, metastasis and radioiodine resistance have always been the difficulties in the treatment of PTC. It is urgent to find more effective treatment methods, and immunotherapy has become the focus of attention. Researchers have applied the targeting of immunotherapy to PTC patients who are not sensitive to conventional treatment [33–35]. More importantly, studies have shown that the expression of PD-L1 in PTC patients was closely related to lymph node metastasis, suggesting that immunotherapy to inhibit PD-L1 may be a choice for patients with lymph node metastasis [36]. Clinical studies have also obtained data. A case report showed that nivolumab could benefit thyroid cancer with BRAF V600E gene mutation. A patient with BRAF V600E mutation and PD-L1 positive who relapsed from PTC to advanced ATC was treated with vemurafenib combined with nivolumab, and the tumor subsided significantly [37]. Although an increasing number of studies have been conducted on the correlation between immunotherapy and PTC, more in-depth basic research and clinical trials are still needed to determine how to apply IRGs to clinical diagnosis and treatment, to clarify the underlying mechanism of immunity and the progression of PTC and to provide a certain theoretical direction for further using IRGs as new targets for PTC treatment and prognosis. IRGs can be regulated by a variety of TFs, which makes the regulatory network between IRGs and TFs highly complex. By analyzing the TCGA database, we identified IRGs and regulatory TFs in PTC to fully understand various IRGs and provide potential biomarkers for the immunotherapy response and immunotherapy targets.

High-throughput sequencing technology can help us identify various biomarkers that are closely related to patient survival at the genetic level. In this study, PTC data from the TCGA were used for bioinformatics analysis to identify prognostic IRGs and establish an immune-related prognostic model. This model contains eight genes, namely, ULBP2, S100A5, LTF, PLXNA4, FAM3B, GIPR, RORB, and TGFBR3. It has been reported that certain genes in the signature are related to the formation and regulation of tumor progression. ULBP2 is reported to bind to the NKG2D receptor on NK cells, trigger the release of various cytokines and chemokines, and promote the recruitment and activation of NK cells [38]. However, pancreatic cancer cells can secrete ULBP2 and reduce the cytotoxicity of NK cells, thereby mediating immune escape and promoting tumor progression, and multivariate regression analysis indicated that ULBP2 was an important independent factor related to poor overall survival. ULBP2 may influence the survival of pancreatic cancer patients [39]. A prospective study showed that ULBP2 expression in the peritoneal fluid of women with endometriosis was significantly high and was related to disease severity [40]. TGFBR3 is a vital part of TGF-β signaling and is usually used as a coreceptor with other members of the TGF-β receptor superfamily. Orthotopic inoculation experiments have shown that the loss of TGFBR3 promotes metastasis via TGF-β-dependent and -independent pathways in renal cell carcinoma cells. Low TGFBR3 expression is correlated with a poor prognosis in renal cell carcinoma patients [41]. Similar results have been reported in head and neck squamous cell carcinoma patients. TGFBR3 can act as a tumor suppressor to hinder tumor progression [42, 43]. Another study showed that macrophage-derived exosomal miR-501-3p can inhibit the expression of the tumor suppressor TGFBR3 and promote the development of pancreatic ductal adenocarcinoma, providing a new target for the molecular therapy of pancreatic ductal adenocarcinoma [44]. Because the functions and mechanisms of some IRGs in the prognostic model have not been reported in PTC, their roles need further research and exploration. We illustrated that the eight-gene prognostic model can be used as an indicator of the immunotherapy response in PTC patients.

The PPI network can be used to identify key node genes. Moreover, univariate Cox regression was carried out to screen prognosis-related IRGs. LTF was identified as a key prognosis-related IRG based on a combination of the PPI network and univariate Cox regression analysis; thus, LTF may be involved in PTC progression. LTF is an iron-binding protein and plays an irreplaceable role in the nonspecific immune system. It is famous for its inherent and adaptive immune function. The protein has been found to have antimicrobial, antiviral, antifungal and antiparasitic activities [45]. Interestingly, it has been found in recent years that LTF also impacts tumor progression. In various cancers, LTF is genetically or epigenetically inactivated. Chen et al. indicated that the expression level of LTF is significantly reduced in thyroid cancer patients and may affect the pathological progression of thyroid cancer based on large-scale data mining [46]. In prostate cancer cell lines, hypermethylation occurs in CpG islands that span the transcription initiation site of LTF. Moreover, through hypermethylation, LTF silencing during the development of prostate cancer supports the role of LTF as a tumor suppressor gene [26]. Tumor-associated macrophages have strong immunosuppressive activity, similar to M2-polarized cells, and play a crucial role in the progression of cancer. Therefore, converting tumor-associated macrophages into a proinflammatory M1-like phenotype is an extremely promising direction for antitumor immunotherapy. Studies have shown that the human LTF immunocomplex can convert tumor-associated macrophages from M2 to M1, and M1-specific markers, which can exhibit strong killing ability in vitro, are significantly increased. In vivo experiments have also proved that the human LTF immunocomplex can significantly promote the accumulation of M1-like macrophages and prolong the survival time of mice. This finding shows that LTF is a promising immunotherapy target [47]. Downregulation of LTF can be found in multiple cancers, including triple-negative breast cancer, nasopharyngeal carcinoma, and renal clear cell carcinoma. The downregulation of LTF is accompanied by tumor growth, invasion and metastasis [25, 48, 49].

However, this study has some limitations. First, since all samples in this study were collected retrospectively, the potential bias associated with unbalanced clinicopathological features of treatment heterogeneity cannot be ignored. Second, the lack of another external validation set is a limitation to our study. Third, the eight-gene model was built and verified using data from the TCGA, which is a public database. It is necessary to provide more prospective data to verify the clinical value of our eight-gene model. In addition, in vivo and in vitro basic and clinical studies are needed to verify and extend these results.

## Conclusion

In summary, we identified an eight-IRG prognostic signature associated with the progression of PTC by performing a series of bioinformatics analyses, and the risk model can be considered an independent prognostic molecular marker to predict the survival of PTC patients. Additionally, by combining the PPI network and univariate Cox regression analysis, a key IRG related to prognosis that may be involved in the progression of PTC was identified. The results of our study will be of great importance in elucidating the potential molecular biological mechanism of PTC and developing new prognostic markers and molecular targets.

## Data Availability

The datasets supporting the findings of this study are available in the from The Cancer Genome Atlas (TCGA) (https://portal.gdc.cancer.gov/), ImmPort (https://www.immport.org/shared/genelists) and Cistrome (http://cistrome.org/CistromeCancer/CancerTarget/) databases.

## Abbreviations

PTC: Papillary thyroid carcinoma
TCGA: The Cancer Genome Atlas
IRG: Immune-related gene
TF: Transcription factor
PFI: Progression-free interval
DEG: Differentially expressed gene
TIC: Tumor-infiltrating immune cell
LASSO: Least absolute shrinkage and selection operator
PPI: Protein–protein interaction
GO: Gene Ontology
KEGG: Kyoto Encyclopedia of Genes and Genomes
ROC: Receiver operating characteristic
AUC: Area under the curve
PCA: Principal component analysis
GSEA: Gene set enrichment analysis
ssGSEA: Single-sample gene set enrichment analysis
PD-1: Programmed cell death 1
PD-L1: Programmed cell death 1 ligand 1 (also called CD274)
CTLA-4: Cytotoxic T lymphocyte associated antigen 4
LTF: Lactotransferrin
DNMT1: DNA methyltransferase 1
ULBP2: UL16 binding protein 2
S100A5: S100 calcium binding protein A5
PLXNA4: Plexin A4
FAM3B: FAM3 metabolism regulating signaling molecule B
GIPR: Gastric inhibitory polypeptide receptor
RORB: RAR related orphan receptor B
TGFBR3: Transforming growth factor beta receptor 3
